# The Greatest and Best

**Published:** 1869-08

**Authors:** 


### THE GREATEST AND BEST.

"By their fruits ye shall know them." "Judge not that ye be not judged." These divine sentences compel us to interpret every good deed as originating from a good motive, and the doer thereof has a right to our admiration, our respect, and our prayers. Who then will not join in a hearty "God bless you," for Stewart and Peabody, the grandest men in the world's history, for their princely benevolence, giving their treasures by millions to bless the worthy, struggling and unfortunate poor. Mr. Stewart is erecting a building in this city which shall furnish a comfortable and elegant home for young women who have to earn a living by their own exertions, at the bare cost of furnishing the necessary food, and to encourage economy, each one pays only for what they order. Every room is tiny, cozy and well warmed and lighted, and no rent is charged for it beyond the actual wear and tear and the cost of the food. This building costs about three millions of dollars.

On Fifty-eighth street and Fifth Avenue, this same gentleman has purchased a large plot of land overlooking the Central Park, which is to be a free home for poor old ladies; and not stopping here, he has selected seven thousand acres of land near this city to be divided into small lots by streets, on each lot a complete home is to be built, to be rented for the interest on the actual cost to such young families as may best live in the country, while the father is engaged in business in town; this enterprise will cost millions more. Who shall not say of this man, that he is the prince of philanthropists; who can number the affectionate prayers that shall go up for him during his lifetime, and the loving remembrance of him by grateful hearts down along the line of the ages yet to come? Let our Vanderbilts and Astors rise to an equal grandeur, and "go and do likewise, while it is day," that grateful generations yet unborn may rise and call them blessed.

